# The effect of COVID-19 on foreign direct investment inflows: stylised facts and some explanations

**DOI:** 10.1186/s43093-022-00129-5

**Published:** 2022-07-07

**Authors:** Imad A. Moosa, Ebrahim Merza

**Affiliations:** Department of Economics, Kuwait University, Shaddadiya, Kuwait

**Keywords:** COVID-19, Foreign direct investment, Crises

## Abstract

The COVID-19 pandemic has impacted every aspect of our lives. The economic effects include the adverse consequences for economic growth, international trade, and foreign direct investment. This paper presents stylised facts about the fall and rebound of FDI inflows as a result of the pandemic. The effects of COVID-19 are considered from three angles: macroeconomic shocks to the economy, theories of foreign direct investment, and studies of the economic effects of disasters and crises. The change of heart away from globalisation and deindustrialisation may discourage FDI flows, which have already been undermined by other factors such as the digitisation of the economy and the emergence of Industry 4.0.

## Introduction

The coronavirus disease 2019 (COVID-19) pandemic has impacted the world in ways that have not been experienced in generations, even though the pandemic is one of the recurring crises and disasters. It is unquestionably one of the most significant global events in recent history, affecting every aspect of daily life. In October 2020, a joint statement by four international organisations (International Labour Organisation, Food and Agriculture Organization, International Fund for Agricultural Development, and the World Health Organization) described the economic and social disruption caused by the pandemic as “devastating” [52]. The pandemic struck at a time when the legacy of the global financial crisis, and the subsequent great recession, were still weighing on the balance sheets of the public and private sectors and when people were still suffering from the consequences of the austerity measures adopted by most countries.

COVID-19 is not only a public health crisis, as it has also severely affected the global economy and financial markets. Among the consequences of the disease mitigation measures implemented by countries around the globe are significant reductions in incomes, higher unemployment rates, and disruptions in the transportation, service, and manufacturing industries. Financial markets worldwide have been affected by the pandemic. Initially, stock markets declined sharply in response to the pandemic, but recovered subsequently in a spectacular manner, buoyed by expansionary monetary policy and later on by the advent of vaccination [30]. The reaction of the gold market to the pandemic was rather unorthodox, as the proposition that gold is a hedge for stock portfolios was not supported for failure to observe negative correlation (for example, [35]). In general, the pandemic had a positive effect on bitcoin prices, even though they remained highly volatile (see, for example, [6]). The main reason for the positive effect of the pandemic on the price of bitcoin was the search by investors for higher yield as policy changes triggered by the pandemic reduced significantly yields on fixed income securities.

On the international scene, the pandemic has hit trade and capital flows. In particular, foreign direct investment (FDI) flows have fallen sharply and disproportionately to the decline in domestic economic activity and trade flows. This is the issue under consideration in this paper where we examine the facts and figures and make an attempt to provide some explanations for the sharp decline in FDI inflows in 2020. The explanations are based on the macroeconomic consequences of the pandemic, theories of FDI, and on the prevailing understanding of the economic effects of crises and disasters. We start with some background notes on the theory and empirics of FDI.

## Background

Foreign direct investment (FDI) is the process whereby the residents of one country (the source country) acquire ownership of foreign assets for the purpose of controlling the production, distribution, and other activities of a firm in another country (the host country). The International Monetary Fund’s *Balance of Payments Manual* defines FDI as “an investment that is made to acquire a lasting interest in an enterprise operating in an economy other than that of the investor, the investor’s purpose being to have an effective voice in the management of the enterprise”. The 1999 *World Investment Report* published by the United Nations Conference on Trade and Development [43] defines FDI as “an investment involving a long-term relationship and reflecting a lasting interest and control of a resident entity in one economy (foreign direct investor or parent enterprise) in an enterprise resident in an economy other than that of the foreign direct investor (FDI enterprise, affiliate enterprise or foreign affiliate)”. The term “long-term” is used in the last definition in order to distinguish FDI from portfolio investment, which is characterised by being short-term in nature and involving a high turnover of securities.

Foreign direct investment FDI has assumed increasing importance over time, becoming a prime concern for policy-makers and a trendy debateable topic for economists. Iqbal et al. [22] suggest that FDI “has become the necessity of every nation as it does accelerate growth in an economy”, describing it as a “double edge weapon as it cuts both ways”, in the sense that “it supplements the resources of the host country and also may replace resources in the host country”. The provision of incentives and the adoption of FDI-stimulating policies are motivated by the realisation that FDI is a more reliable source of capital than portfolio investment. This lesson has been learned from the Asian crisis of the 1990s. Lipsey [27], for example, argues that FDI has been the least volatile source of international investment for host countries, with the notable exception of the USA. He also argues that FDI has been the most dependable source of foreign investment for developing countries.

Chakrabarti [8] attributes interest in FDI to its rapid growth, particularly in the 1990s (see [44]), and its importance for developing countries as a viable alternative to capital markets. In addition to the rapid growth of FDI, Moosa [31] attributes interest in FDI to (1) the concern it raises about the causes and consequences of foreign ownership; (2) its importance as a source of capital for developing countries; and (3) the role it plays in the transformation of the former communist countries. According to the World Investment Report of the UNCTAD [44], 208 changes in FDI laws were made by 71 countries in 2001. Of these changes, 194 (93 per cent) created a more favourable climate in an effort to attract more FDI.

The literature on FDI is huge and still growing. In what follows, a brief mention of some of the contributions to this literature is presented. A notable issue is corruption as a determinant of FDI inflows. This issue is examined by Hasan et al. [17] who argue that FDI is significantly related to corruption, even though the effect could be positive or negative in accordance with the helping hand and grabbing hand theories, respectively. The determinants of FDI inflows in general have been dealt with extensively. For example, Iqbal et al. [23] point out that plausible determinants include market size, inflation, trade openness, and current account balance. FDI as a promoter of growth is an issue that has received considerable attention in the literature (for example, [20]). The connection between FDI, trade, and official development assistance is undertaken, among others, by Iqbal et al. [21]. The most recent issue of FDI under COVID-19, which is the issue examined in this paper, is considered by Yadav and Iqbal [53] who explore the socio-economic scenarios for the South Asian region before and after the outbreak of the pandemic.

## Methods

The objective of this paper is to examine the effect of the COVID-19 pandemic on FDI inflows. As at the middle of 2022, the pandemic is still going on and data availability is limited, which precludes the possibility of presenting a full-fledged and formal statistical analysis. As a result, this paper is based on descriptive analysis of the stylised facts with reference to economic theory.

The starting point is to present the stylised facts pertaining to the behaviour of FDI inflows, which is done in the following section. The stylised facts are based on the data reported by the Organisation for Economic development and Co-operation and Development (OECD). Even though this paper is descriptive, dome forecasts will be presented for the rebound in FDI inflows based on four different scenarios. Once the stylised facts have been presented, the effects of COVID-19 are considered from three angles: macroeconomic shocks to the economy, theories of foreign direct investment, and studies of the economic effects of disasters and crises.

## Observations

**Fig. 1:**
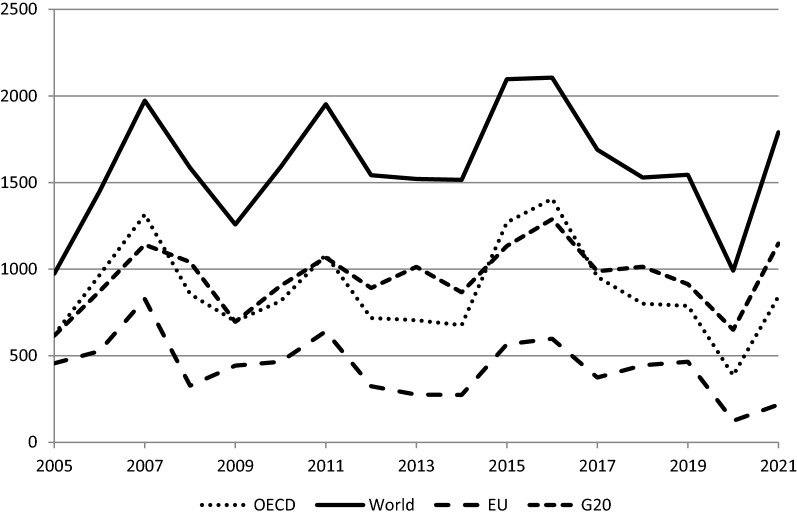
FDI inflows (OECD data, billion dollars)

**Fig. 2:**
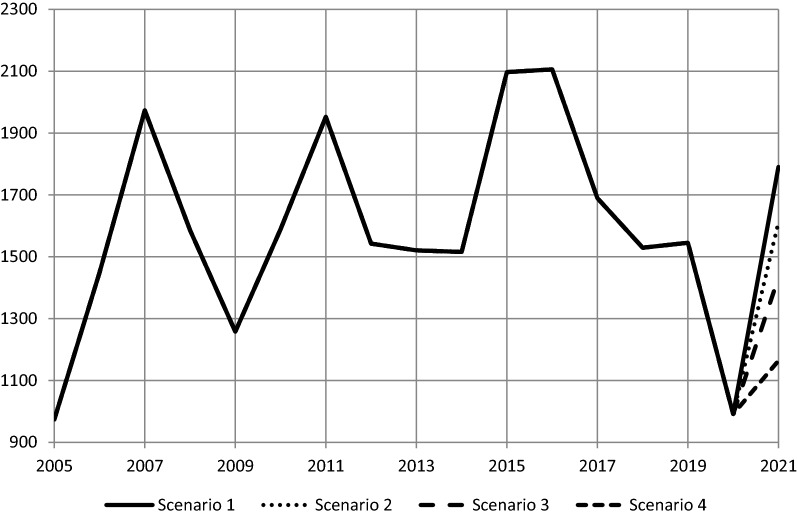
Scenarios for the 2021 rebound in World FDI inflows

Figure 1, which covers the period up to the first half of 2021, shows FDI inflows (in billion dollars) according to the OECD data [34]. In 2020, world FDI inflows declined by 36%, but the decline was more pronounced in the European Union where inflows went down by 73%, which is a bigger decline than what was witnessed by OECD countries in general (51%). In 2021, however, FDI inflows rebounded as indicated by the available figures for the first two quarters of the year. If the rebound witnessed in the first half of the year had continued, this would have taken FDI inflows to a higher level than in 2018. If this were the case, the world FDI inflows should have risen by 81%, whereas the corresponding figure for OECD countries would have been 118%. However, it is unlikely that the growth rate of FDI inflows would have continued to grow at the same rate for the whole year, particularly with the uncertainty created by the emergence of the Omicron variant of the Coronavirus. This is why Fig. 2 shows four scenarios for the rebound in FDI inflows, where the most optimistic scenario (scenario 1) is that growth in the second half would have continued at the same pace as that of the first half. In the other three scenarios, the second half growth would have been a fraction of growth in the first half.

According to UNCTAD [47], FDI recovery will be uneven, predicting that developed economies are set to drive global growth in FDI, both because of strong cross-border mergers and acquisitions (M&A) activity and large-scale public investment support. However, prospects are highly uncertain and will depend on, among other factors, the pace of economic recovery and the possibility of pandemic relapses, the potential impact of recovery spending packages on FDI, and policy pressures. Another factor is lingering uncertainty about access to vaccines, the emergence of virus mutations, and the reopening of economic sectors.

In Fig. 1, we can see that FDI inflows do not exhibit any secular trend, but rather they move in cycles (this has been the case at least since 2005). Cyclical peaks occurred in 2007, 2011, and 2016, whereas cyclical troughs can be seen in 2009, 2014, and 2020. It can be readily recognised that the two troughs of 2009 and 2020 are associated with the global financial crisis and the COVID crisis, respectively. Kalotay and Sass [25] suggest that the 2020 fall in FDI was the result of new developments that started well before the advent of the pandemic, and that the pandemic aggravated the situation to create what they call a “perfect storm”. This is why they believe that COVID was not a “game-changer” for FDI in terms of jump-starting fresh trends in FDI flows. The new developments include digitalisation and the emergence of Industry 4.0, which make the operations of multinational corporations (MNCs) more intangible and less dependent on investment in physical assets.^1^ Another contributory factor is the growing imperative for sustainable development, as it has become apparent that sustainability may be incompatible with the maximisation of FDI flows. Yet another development is the fragmentation of international trade and investment policy making, reflecting protectionist and populist pressures. The pandemic has actually encouraged “deglobalisation” and “slowbalisation”.

The UNCTAD [46] suggests that reshoring, diversification, regionalisation, and replication can lead to diminishing cross-border investment. Reshoring is expected to lead to shorter, less fragmented supply chains and geographical concentration of value added, primarily in higher-technology industries.^2^ This trajectory may lead to more divestment and a shrinking pool of efficiency-seeking FDI. Diversification, which will lead to a wider distribution of economic activities, will primarily affect services and the global value chain (GVC)-intensive manufacturing industries. Reliance on supply chain digitalisation may cause those GVCs to be more loosely governed, platform-based and asset-light. Regionalisation will reduce the physical length but not the fragmentation of supply.^3^ Replication is expected to lead to shorter supply chains, a rebundling of production stages, and consequently to more geographically distributed activities and more concentrated value added. This trajectory implies a shift from investment in large-scale industrial activity to distributed manufacturing, which relies on lean physical infrastructure and high-quality digital infrastructure.

According to UNCTAD [47], the 2020 decline in FDI inflows was heavily skewed towards developed economies, where they fell sharply by 59% to $329 billion, a level that was last seen in 2003. FDI flows to Europe fell by 78%, largely because of negative FDI in countries with significant conduit flows, such as the Netherlands and Switzerland. FDI to North America declined somewhat less sharply, by 42%. FDI to developing economies declined at a more moderate rate of 9%, mainly because of robust flows in Asia. The fall in FDI inflows across developing regions was uneven, with 45% in America and 1% in Africa. In contrast, flows to Asia rose by 3%.

**Fig. 3:**
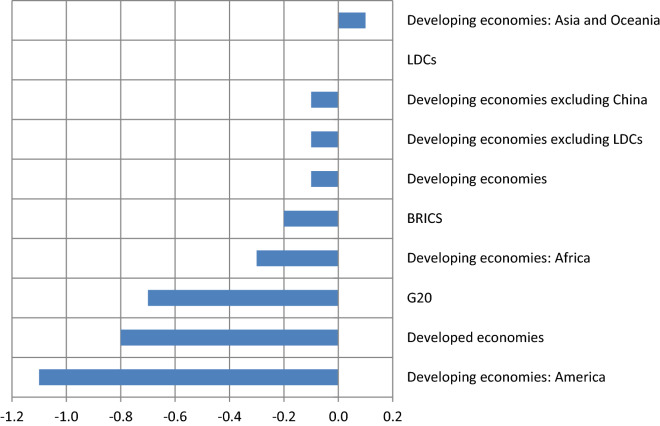
Change in ratio of FDI inflows to GDP (2020)

Figure 3 shows changes (in percentage points) of the ratio of FDI inflows to gross domestic product (GDP) in various country groups according to the data found in UNCTAD [48]. The only country group that witnessed an increase in the ratio of FDI inflows to GDP is the group of developing countries from Asia and Oceania. Developing economies in the Americas were more badly affected than developed countries. According to the UNCTAD [47], COVID-19 has also caused a collapse in investment flows to sectors relevant for the Sustainable Development Goals (SDGs) in developing countries.^4^ FDI inflows to infrastructure fell by 54%, and the same goes for health. The most affected sector, which experienced a decline of 67%, was the provision of water and sanitation to industry and households. FDI inflows for food and agriculture went down by 49%.

**Fig. 4:**
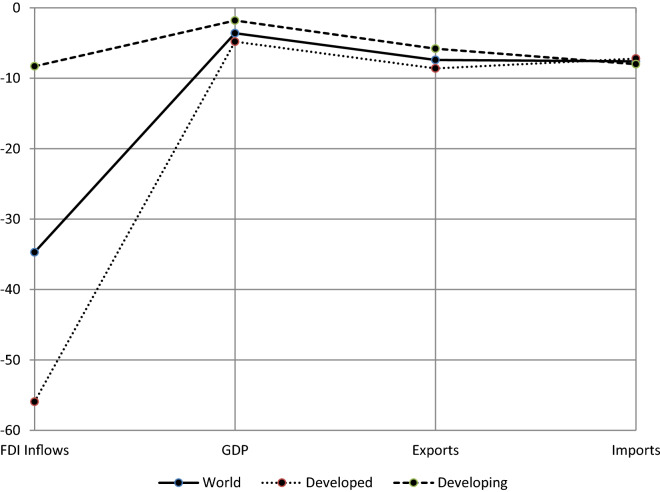
A comparison of the growth rates of FDI inflows, GDP, and trade (2020)

In Fig. 4, we can see a comparison between the rates of decline in 2020 of FDI inflows, GDP, exports, and imports [48–50]. As we can see, FDI inflows fell more sharply than either GDP or trade flows. The UNCTAD [47] notes that FDI flows react more strongly to crises than trade and GDP and take both more time and more (policy) effort to recover. This observation indicates some sort of a delink between trade and FDI flows when FDI can be viewed as an alternative to trade. Kalotay and Sass [25] put forward various possible explanations for the sensitivity of FDI to the effects of the pandemic. One explanation is that COVID-19 reinforced the pre-existing trends that would have affected the growth of the volume of FDI adversely, even without the crisis. Still, the difference between the decline in international trade and FDI is greater than what can be justified by this proposition. A more plausible explanation that they present is that FDI creates productive assets while trade is typically a one-off transaction. If FDI stops, production still continues with the pre-existing assets but if trade stops, the economy, or large parts thereof, stops. They also note that FDI was instantly struck by lockdown, stoppage, and border closing measures in the early weeks of 2020.

## Results

The descriptive analysis presented in this paper shows that if the rebound in FDI inflows witnessed in the first half of 2020 had continued, this would have taken FDI inflows to a higher level than in 2018. However, it is unlikely that FDI inflows would have continued to grow at the same rate for the whole year, particularly with the uncertainty created by the emergence of the Omicron variant of the Coronavirus. This is why four scenarios are presented for the rebound of FDI inflows, such that the most optimistic scenario (scenario 1) is that growth in the second half would have continued at the same pace as that of the preceding half. In the other three scenarios, the second half growth would have been a fraction of growth in the first half.

The analysis shows that FDI inflows do not exhibit any secular trend, but rather they move in cycles (this has been the case at least since 2005). Two troughs occurred in 2009 and 2020 as a result of the global financial crisis and the COVID crisis, respectively. The analysis indicates that the change in trend started well before the advent of the pandemic, but the pandemic augmented the bearish outlook for FDI inflows. The change in trend can be attributed to digitalisation and the emergence of Industry 4.0. Furthermore, the fragmentation of international trade and investment policy making, ha also appeared as a contributory factor.

The analysis reveals that the outbreak of COVID-19 caused a slowdown of the capital expenditures of MNCs, forcing them to close some production locations or to operate at lower capacity. Apart from the primary effect of delayed capital expenditures, the pandemic exerted an indirect effect that worked through lower profits in foreign affiliates, leading to shrinking reinvested earnings.

The analysis of the effect of the pandemic as postulated by the theories of FDI reveals that some theories are more relevant and provide better explanations than others. Some of the theories turn out to be totally irrelevant. The relevant theories, which can be used to explain the effect of the pandemic on FDI inflows, include the differential rates of return hypothesis, the portfolio diversification hypothesis, and the market size hypothesis. In relation to market size, the analysis does not reveal one-to-one correspondence between changes in GDP and changes in the ratio of FDI to GDP, but a positive relation can be seen clearly. The available data shows that in 2020, developed countries suffered a 4.8% decline in the size of their economies and lost a 0.8 percentage point of FDI relative to GDP.

The results also confirm the relevance of the internal financing hypothesis. Based on available data, the capital expenditures of many MNCs slowed down while most of the top 100 MNCs reported lower profits, which translated into lower reinvested earnings. Significant cross-regional variation can be observed in earnings revision and the share of reinvested earnings in FDI.

## Discussion: macroeconomic shocks

In a classic recession, aggregate demand falls short of aggregate supply (a situation of deficient demand), which prompts policy-makers to fill the gap in the supply–demand balance, typically via fiscal expansion. The COVID recession, however, is more complicated because it involves both supply and demand shocks. Hence, the macroeconomic effects of COVID-19 can be examined by distinguishing between the supply-side and demand-side effects of the pandemic. A supply shock is anything that reduces the economy’s capacity to produce goods and services at given prices. Lockdown measures preventing workers from going to work represent a supply shock, and so do absenteeism and interruption to international trade. A demand shock, on the other hand, reduces the ability or willingness of consumers to purchase goods and services at given prices. For example, a demand shock (to the hospitality sector) occurs when people avoid restaurants for fear of being infected. The financial effects of COVID-19 include the effects on corporate debt, stock and commodity markets, and the financial sector in general. The effects can also be seen in terms of disruptions to the circular flow of income, which depicts flows of money, goods, and services between various sectors of the economy (see, for example, [30]). With respect to trade and FDI, the effects (as portrayed by the circular flow of income) arise from disruptions occurring between firms and the rest of the world, characterised by diminished exports, imports, and payments for supply chain transactions.

Naturally, the effect of COVID-19 is uneven, as negative demand shocks are concentrated in the economies most severely hit by the pandemic. Effects caused by production stoppages and supply chain disruptions were felt particularly in economies that are closely integrated in the global supply chains centred around China, Korea, and Japan, as well as South-East Asian economies. One would expect the effect on FDI inflows to have a greater impact in those countries that have been forced to take the most drastic measures to contain the spread of the virus.

The outbreak of COVID-19 caused a slowdown of the capital expenditures of MNCs and their foreign affiliates. Some production locations were closed or operated at lower capacity, implying that MNCs temporarily halted fresh investment in physical assets and forced them to delay expansion. The COVID-caused macroeconomic shocks were consequential for three kinds of FDI: market-seeking, efficiency-seeking, and resource-seeking.^5^ Apart from the primary effect of delayed capital expenditures, a further (indirect) mechanism works through lower profits in foreign affiliates, leading to shrinking reinvested earnings. In the economies most affected by COVID-19, reinvested earnings make up about 40% of total FDI inflows. We will come back to this point later.

## Discussion: the effect of COVID-19 in terms of the theories of FDI

Nawo and Njangang [32] argue that the effect of the COVID-19 outbreak on FDI can be examined by considering FDI theories under uncertainty, because the pandemic has brought with it a high level of uncertainty. They consider two groups of theories: micro (industrial organisation) theories and macro (cost of capital) theories. Theories of FDI may be classified under the following headings: (1) theories assuming perfect markets, (2) theories assuming imperfect markets, (3) other theories, and (4) theories based on other factors and considerations (for details, see [31]. Only some of the theories of FDI can be used to explain the decline in FDI inflows as a result of the pandemic.

The differential rates of return hypothesis postulate that capital tends to flow from countries with low rates of return to countries with high rates of return in a process that eventually leads to the equality of ex ante real rates of return. This means that a country experiencing a lower rate of return caused by the pandemic (for example, as a result of shrinking sales) would attract less FDI inflows. The portfolio diversification hypothesis is similar to the differential rates of return hypothesis, except that it considers both return and risk. When the assumption of risk neutrality is relaxed, risk becomes another variable upon which the FDI decision is made. If risk is associated with the stringency of the restrictions imposed to combat the disease, one would expect a bigger decline in FDI in countries producing less favourable risk-return combinations. Nawo and Njangang [32] consider COVID-19 as producing economic uncertainty with respect to the return on investment.

**Fig. 5:**
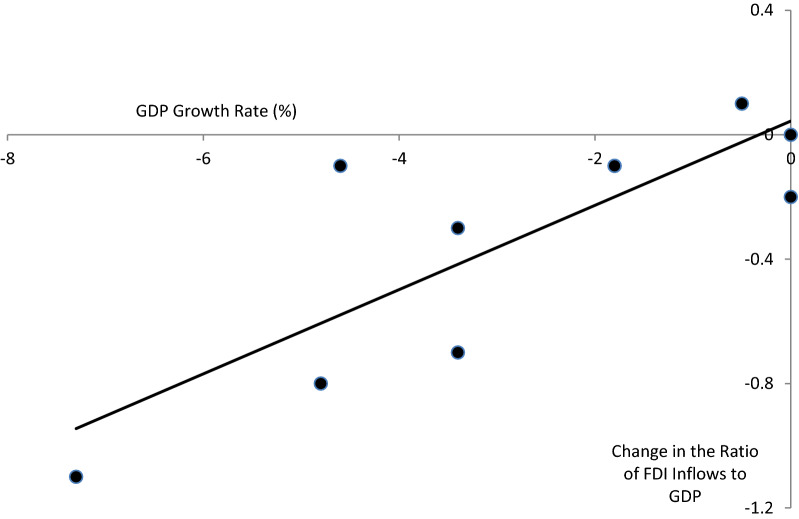
Relation between GDP growth and change in the ratio of inflows to GDP

Another relevant theory is the market size hypothesis, which states that the volume of FDI in a host country depends on its market size, which is measured by the sales of a multinational corporation (MNC) in that country or by its GDP (that is, the size of the economy). As soon as the size of the market of a particular country has grown to a level warranting the exploitation of economies of scale, the underlying country becomes a potential target for FDI inflows. The relationship between FDI and output can be derived from neoclassical models of domestic investment. The rationale for the hypothesis that firms invest more in response to growth in sales is based on neoclassical domestic investment theories. In Fig. 5, we can observe a positive relation between the GDP growth rate and the change in the ratio of FDI inflows to GDP [48, 49] for ten country groups (the same country groups as in Fig. 3). There is no one-to-one correspondence between changes in GDP and changes in the ratio of FDI to GDP, but a positive relation can be seen clearly. In 2020, developed countries suffered a 4.8% decline in the size of their economies and lost a 0.8 percentage point of FDI relative to GDP.

The internal financing hypothesis is also relevant where internal financing refers to the utilisation of the profit generated by a subsidiary to finance the expansion of FDI by an MNC in the same country where the subsidiary operates. This hypothesis postulates that MNCs commit a modest amount of their resources to initial direct investment, while subsequent expansions are financed by re-investing the profits generated from operations in the host country. The hypothesis, therefore, implies the existence of a positive relation between internal cash flows and investment outlays, which is plausible because internal financing is cheaper than external financing.

If we look at the facts and figures, we can see how relevant this hypothesis is. The UNCTAD [45] reported that more than two-thirds of the top 100 MNCs had issued statements on the impact of COVID-19 on their business. As a result, the capital expenditures of many of them slowed down in the affected areas. Most of the top 100 MNCs also reported lower profits, which would translate into lower reinvested earnings. According to UNCTAD [45] the top 5000 MNCs, which account for a significant share of global FDI, have seen downward revisions of the 2020 earnings estimates of 9% due to COVID-19. Hardest hit were the automotive industry (− 44%), airlines (− 42%), and energy and basic materials industries (− 13%). Earnings revision and the share of reinvested earnings in FDI vary from one part of the world to another. For the whole world, average earnings revision was − 9% whereas the share of invested earnings in FDI is 52%. In transition countries, where 93% of earnings are reinvested, earnings revision was − 10%.^6^

Last, but not least, we have a hypothesis linking FDI inflows to political risk and country risk where the latter encompasses the former, as it takes into consideration political, economic, and other factors, such as natural disasters. What is under consideration here is the possibility of adverse economic or political decisions, including changes in the “rules of the game”, such as the possibility of raising the level of taxes or imposing restrictions on profit repatriation. Under COVID-19, there is a higher probability of changing the rules of the game in countries hardest hit by the pandemic (for example, by imposing severe restrictions, such as a total travel ban).

## Discussion: the effect of COVID-19 as a crisis and a disaster

Literature is available on the effect of natural disasters on FDI. Few studies have been conducted on the specific issue of the COVID crisis (disaster) on FDI. Anuchitworawong and Thampanishvong [2] explore the effect of natural disasters on FDI in Thailand and produce results indicating that higher severity of natural disasters, captured by their constructed composite index, tends to reduce FDI flows into Thailand. Escaleras and Register [13] analyse the linkage between FDI and the number of disasters and find that natural disasters are negatively and significantly associated with FDI inflows.

The effect of health-related disasters on FDI has been considered by several authors. Asiedu et al. [3] present a model of the effect of the human immunodeficiency virus/ acquired immunodeficiency syndrome (HIV/AIDS) on FDI and test it on a panel of data covering 84 developing countries over the period 1990 to 2008. They reveal that HIV/AIDS has a negative effect on FDI. By using annual panel data of 70 developing countries from 1985 to 2004, Azemar and Desbordes [5] find that in the absence of HIV and malaria, net FDI inflows in the median Sub-Saharan Africa could have been one-third higher during the period 2000–2004, with slightly more than one-half of this deficit explained by malaria. Alsan et al. [1] analyse the effect of public health on gross inflows of FDI by using panel data on 74 industrialised and developing countries over the period 1980–2000. Their main finding is that gross inflows of FDI are strongly and positively influenced by population health in low- and middle-income countries. Their empirical results suggest that, after controlling for other relevant variables, raising life expectancy by one year boosts gross FDI inflows by 9%.

The effect of the COVID pandemic on FDI flows has been examined by some economists. Hayakawa and Mukunoki [18] produce results that suggest the following: (i COVID-19 had negative effects on the trade of importing and exporting countries; (ii these effects have been insignificant since July 2020; and (iii COVID-19 has had heterogeneous effects across FDI-industries. Focussing on China’s FDI, Fang et al. [14] found that the numbers of new confirmed cases, new deaths, and cumulative confirmed cases have significant negative impacts on FDI. By using monthly bilateral FDI data, Fu et al. [15] found that (1) the outbreak reduced both the extensive and intensive margins of FDI; (2) the mortality rate reduced FDI margins; (3) FDI was more sensitive to the pandemic situation in host countries for both OECD and emerging countries; and (4) the service sector’s FDI was more severely affected by the pandemic than other sectors’ FDI. Nawoa and Njangang [32] examine the effect of the pandemic on FDI in 79 developed and developing countries and obtain results indicating that both the total number of deaths and cases are negatively correlated with FDI.

Kalotay and Sass [25] argue that not learning from the previous crises would be a mistake for various reasons and suggest a strong rationale for analysing and comparing at least the major crises affecting a large part of the world economy. Crises have in common the disruption of MNCs operations, making business as usual difficult or impossible and putting pressure on them to react by adjusting their operations. In response to a crisis, MNCs may react by curtailing activities, whereas smaller firms may go out of business. The effects of crises on FDI have been examined repeatedly, producing a large set of findings.

In past crises, FDI was typically found to be more stable and resilient than other financial flows (particularly portfolio investment) due to its link with productive capacities, and the inherent fixed and sunk costs. This is to be expected, since it is easier and quicker to liquidate a stock portfolio than to unwind a physical business or close down a factory. The UNCTAD [47] notes that FDI flows are more stable and resilient than other international financial flows and external sources of finance for developing countries (such as portfolio flows and bank loans). These studies have also revealed that FDI was affected more than macroeconomic variables, such as GDP and trade flows. These results can be found in Thompson and Poon [41], Athukorala [4], Doraisami [10], and Thangavelu et al. [39] for the Asian crisis and in Vintila [51] and Lund et al. [28] for the global financial crisis.

A consensus view, on which crisis has had the biggest and the most lasting impact on FDI, does not exist. In general, the available evidence shows that the global financial crisis, by its financial nature, was the deepest and the longest crisis so far (for example, [7, 37, 51]. Nevertheless, the OECD [33] thinks that the COVID crisis could also become the longest and deepest recession (for more on a comparison between the global financial crisis and the COVID crisis, see [30]. Since recessions lead to shrinking FDI flows, the effect on FDI becomes apparent. A related issue is that of how long it takes FDI flows to return to their pre-crisis level. In the case of the Asian crisis, some studies show FDI recovery (for example, [19, 42] whereas others show declining FDI inflows into South-East Asia in the post-crisis period despite output growth [39]. We have already seen from the stylised facts that FDI inflows have rebounded, probably reaching pre-pandemic levels in 2021.

The literature shows that the Asian crisis brought about sectoral changes in FDI in the direction of export-oriented activities [12, 39]. In Eastern Europe, the global financial crisis brought about a shift towards higher value-added activities and non-financial services, as shown by Kalotay [24]. Country-level studies of the COVID crisis reveal structural changes in favour of agri-food, machinery, pharma, and logistics (for example, [26]). The UNCTAD [47] notes that FDI downturns can presage a shift in sectoral patterns and types of investment.

Cross-border mergers and acquisitions (M&As) seem to fall less than other forms of international flows as they represent an essential component of corporate restructuring and because of “fire sales”.^7^ It has been highlighted that in the Asian crisis, some of the M&A transactions that were thought to be fire sales were in fact takeovers that saved acquired firms and protected their activities [54]. The trajectory of FDI in crisis may also depend on the motivation of investment. For example, efficiency-seeking investment led recovery in the aftermath of the Asian crisis [12]. Furthermore, the UNCTAD [47] notes that international deal activity (including both project finance and M&As) falls further and takes longer to recover than domestic deal activity.

The UNCTAD [47] makes further observations on the behaviour of FDI during crises. The first is that recovery of investment in lower-income developing countries can take relatively long, due to both their greater reliance on greenfield projects and investors’ more risk-averse behaviour after crises.^8^ On M&As, the UNCTAD notes that in crises, M&As include opportunistic purchases as well as transactions necessary for corporate restructuring. Another observation is that MNCs and their foreign affiliates adjust to crises and recover relatively quickly compared with smaller domestic firms. The UNCTAD also notes that the presence of resilient MNCs in host countries can support faster recovery from crises, depending on linkages with domestic suppliers.

The literature produces mixed results on some issues. For example, Moon et al. [29] believe that FDI and other types of MNC investment contribute to stability and recovery, but Doraisami [10] thinks that FDI could be a source of vulnerability. And while Athukorala [4] shows that FDI facilitated recovery, others show that the recovery of FDI followed economic recovery (for example, [37, 39]). Alternatively, Simionescu [38] found a reciprocal relation between economic growth and FDI. It has also been found that cross-border M&As may play a role in restructuring the economic activities for the post-crisis period [54].

Every crisis has an effect on public policy or gives rise to suggestions of policy changes. For example, Thompson and Poon [41] emphasise the need for reforming investment promotion. Plummer and Cheung [36] focus on the role of investment liberalisation and facilitation (in the aftermath of the Asian crisis). Dornean et al. [11] stress the importance of a supporting regulatory environment. Kalotay [24] emphasises the need for industrial policy supporting the upgrading of activities. The UNCTAD [47] notes that most post-crisis policy interventions have aimed at facilitating or stimulating FDI (rather than restricting it) to support recovery. Moosa [30] demonstrates that the COVID crisis has implications for economic thought and public policy, including a change of heart away from neoliberalism, the necessity of government intervention in economic activity, and that the market is not a magical device that restores equilibrium and prosperity after a shock. Obviously, these implications are consequential for FDI.

## Concluding remarks

The pandemic is likely to force a rethinking of the benefits and costs of globalisation for at least two reasons. The first is that the collapse of supply chains may force companies to look for domestic suppliers to avoid interruption. The second pertains to the importance of the domestic production of medical goods, which has been recognised following the scramble for personal protection equipment and the price gouging events of the early stages of the pandemic. This is why Gray [16] believes that “the era of peak globalisation is over”, describing the impact of the pandemic as “not a temporary rupture in an otherwise stable equilibrium”. He goes as far as saying that “the crisis through which we are living is a turning point in history”. Therefore, he expects a “more fragmented world that in some ways may be more resilient” to come into being.

Likewise, Darius [9] thinks that the pandemic will propel anti-globalist political forces, with a shift towards the era of deglobalisation, suggesting two reasons for the shift. The first is that the pandemic itself adheres to the central narrative that has instigated anti-globalist political movements and policies throughout the world: the vulnerability of the domestic populace to nefarious foreign elements. The second reason for the shift in favour of deglobalisation pertains to the measures that countries have taken in response to the pandemic. For some time now, the prevailing narrative among political leaders in relation to globalisation is that it is the phenomenon of our time that both policy-makers and the electorate have to contend with in all its forms: the good, the bad, and the ugly.

The same goes for deindustrialisation, which some economists and observers believe to be a natural outcome of progress as an advanced economy tends to move away from manufacturing industry towards services. For example, an eminent economist, Jagdish Bhagwati, thinks that those who argue for boosting manufacturing output suffer from “manufacturing fetishism”, suggesting that the service industry is as good as manufacturing in generating jobs and boosting exports [40]. This claim, however, is not supported by simple observable facts such as jobless growth, a state of affairs where high unemployment coexists with expansion in GDP. This state of affairs can be attributed to structural changes in the economy rather than a cyclical recovery—one form of this structural change is deindustrialisation. The change of heart away from globalisation and deindustrialisation will have adverse consequences for FDI, which may not be bad after all.

At this stage, it seems appropriate to ask what we have learned about the implications of the pandemic. Like any crisis, the pandemic represents both a challenge and an opportunity: a challenge because it has caused immense suffering, and an opportunity because it gives us a chance to reflect on what should be done to alleviate suffering when the next crisis hits. The pandemic has had a negative impact on FDI flows but it has also provided an opportunity to reflect on everything, including FDI.

## Data Availability

Available upon request.

## Abbreviations

AIDS: Acquired immunodeficiency syndrome
COVID: Coronavirus disease
FDI: Foreign direct investment
GDP: Gross domestic product
GVC: Global value chain
HIV: Human immunodeficiency virus
MNC: Multinational corporation
OECD: Organisation for Economic Development and Co-operation and Development
SDGs: Sustainable development goals
UNCTAD: United Nations Conference on Trade and Development

## Footnotes

1 Industry 4.0, or the fourth industrial revolution is the trend towards automation in manufacturing technologies and processes, includes cyber-physical systems, industrial Internet of things, cloud computing, cognitive computing, and artificial intelligence. The first industrial revolution (1760–1870) was marked by a transition from hand production methods to machines. The second industrial revolution (1871–1914) was marked by the installations of extensive railway and telegraph networks, as well as the introduction of electricity. The third industrial revolution (or the digital revolution) occurred in the post-war period, propelled by technological advancement such as communication technologies and supercomputers.

2 Reshoring refers to action taken by corporations to move their production facilities back to the home countries because of supply bottlenecks and the pandemic-caused problems associated with global supply chains.

3 Regionalisation is the move from global to regional supply chains.

4 These include eradication of poverty and hunger, alleviation of inequality, and the provision of good healthcare and education, clean water, affordable energy, and resilient infrastructure. It is not clear how FDI contributes to the achievement of these goals. FDI may provide good healthcare and education and clean water, but that will not be cheap for consumers. The provision of affordable energy by multinationals is wishful thinking. And if anything, FDI aggravates inequality.

5 Market-seeking FDI is driven by host market characteristics, such as size, growth, and structure. Resource-seeking FDI is motivated by the desire to have access to cheap resources such as raw materials and labour. Efficiency-seeking FDI is intended to utilise sources of competitiveness and economies of scale and scope.

6 Transition countries are still in the process of moving away from central planning to a market economy. They include former Soviet republics and eastern bloc countries. For some reason, the countries that have joined the European Union have completed transition whereas others (including Russia) are still in transition.

7 A fire sale involves the offering for sale of assets at very low prices by a seller facing bankruptcy.

8 Greenfield and brownfield investments are two types of foreign direct investment. Greenfield investment involves a new venture—for example, by opening a new production facility in the host country. Brownfield investment, on the other hand, involves the purchase or leasing of an existing production facility.
